# Longitudinal dynamics and lineage cross-reactivity of the human antibody response to West Nile virus

**DOI:** 10.1128/mbio.01766-26

**Published:** 2026-09-17

**Authors:** Austin G. Leach, Lingling Xu, Shilu Malakar, Monia Pacenti, Stefania Vogiatzis, Sanjeev Kumar, Jacob Vander Velden, Meng Wang, Mitchell J. Kendzel, Veronika I. Zarnitsyna, Sean Hui, Michael N. Nguyen, Christopher A. Nelson, Tyshawn Ferrell, Christopher J. Neufeldt, Daved H. Fremont, Vineet D. Menachery, Michael P. Busch, Philip J. Norris, Marion Lanteri, Luisa Barzon, Jens Wrammert, Mehul S. Suthar

**Affiliations:** 1 Division of Infectious Disease, Department of Pediatrics, Center for Childhood Infections and Vaccines (CCIV), Emory University School of Medicine 209740, Atlanta, Georgia, USA; 2 Department of Pediatrics, Children’s Healthcare of Atlanta, Emory University School of Medicine, Atlanta, Georgia, USA; 3 Emory Vaccine Center, Emory University School of Medicine 12239, Atlanta, Georgia, USA; 4 Microbiology and Virology Unit, Padova University Hospital 18624 , Padova, Italy; 5 Department of Molecular Medicine, University of Padova 208970, Padova, Italy; 6 Department of Microbiology and Immunology, Emory University School of Medicine 197280, Atlanta, Georgia, USA; 7 Department of Pathology and Immunology, Washington University School of Medicine 12275 , St. Louis, Missouri, USA; 8 Department of Biochemistry and Molecular Biophysics, Washington University School of Medicine 12275, St. Louis, Missouri, USA; 9 Department of Molecular Microbiology, Washington University School of Medicine 12275, St. Louis, Missouri, USA; 10 Vitalant Research Institute 166672, San Francisco, California, USA; 11 Department of Laboratory Medicine, University of California San Francisco 8785, San Francisco, California, USA; Tsinghua University, Beijing, China

**Keywords:** West Nile virus, humoral immunity, antibody, neutralization, human samples, durability

## Abstract

West Nile virus (WNV) is an emerging/re-emerging flavivirus representing one of the major global causes of mosquito-borne encephalitis, yet no approved therapeutics or vaccines currently exist. To characterize the kinetics of WNV-specific immunity, we evaluated the humoral response in a longitudinal cohort of 55 WNV lineage 1-infected blood donors up to 1 year. We demonstrate that WNV infection elicits robust and durable binding and neutralizing antibodies. Using a biphasic decay model, we estimated the half-life of neutralizing antibodies to be 37 days during the rapid decay phase and 472 days during the extended slow decay phase. Notably, the magnitude and durability of these antibodies were largely independent of viral RNA persistence in the whole blood (WB) compartment. To assess the breadth of this response, we evaluated cross-reactivity against an emerging divergent WNV lineage 2 strain and observed that WNV lineage 1-immune sera exhibited reduced neutralizing activity with high variability between donors during both acute and convalescent phases. These deficits in heterologous neutralization were reciprocally confirmed using an independent WNV cohort from Italy. Together, our results establish that lineage-specific divergence results in antibodies with a highly variable reduction in neutralization, indicating that successful WNV vaccine candidates should overcome this variability to confer pan-lineage protection.

**IMPORTANCE:**

West Nile virus (WNV) is a growing global health threat spread by mosquitoes that can cause severe disease, yet there are currently no approved vaccines or treatments available. We followed 55 WNV lineage 1-infected blood donors up to 1 year to study their antibody response to infection. We found that natural infection generates a long-lasting antibody response. Interestingly, the magnitude and durability of these antibodies were maintained regardless of viral RNA persistence in the whole blood compartment. However, a potential gap in cross-protective immunity emerged when we examined WNV lineage 1-specific antibodies against WNV lineage 2, a genetically distinct and divergent viral strain with over 25 mutations in the envelope protein. Sera from WNV lineage 1-infected donors showed a highly variable reduction in neutralizing activity against lineage 2 WNV during both the acute and convalescent phases of infection. We corroborated this observation using an independent cohort of patients from Italy, again demonstrating reduced cross-neutralization between the two viral lineages. Together, our results establish that lineage-specific divergence results in reduced cross-neutralization of heterologous strains, indicating that successful WNV vaccine candidates should overcome this barrier to confer pan-lineage protection.

## INTRODUCTION

Responsible for worldwide outbreaks of mosquito-borne encephalitis, West Nile virus (WNV) continues to remain a significant emerging and re-emerging flavivirus. WNV was first identified in Uganda in 1937 and has been circulating in Europe and the Mediterranean Basin since at least the late 1950s, causing sporadic infections and outbreaks in humans and animals (1). Over the span of 30 years, WNV has spread globally and become endemic in several European countries. Since 1999, WNV has remained a constant threat in North America, including the United States, which has accumulated over 55,000 confirmed cases with a case fatality rate of approximately 5% (2–5). Currently, there are no vaccines or therapeutics approved for use in humans to protect against WNV infection (2, 6).

Phylogenetic analysis of WNV genome sequences has revealed nine evolutionary lineages, but only lineages 1 (WNV1) and 2 (WNV2) have been linked to human disease (7). In the Americas, from Canada to Argentina, all human and animal outbreaks are caused by a strain classified within clade WNV1a, first detected in New York in 1999 (8). This virus was nearly identical to a neurovirulent WNV strain circulating in Israel in 1998. Conversely, several WNV1a strains, distinct from those in the Americas and introduced from African countries, caused large outbreaks in southern Europe and the Mediterranean until the emergence of WNV2 (9). Originally isolated in Sub-Saharan Africa, where it was historically linked to mild febrile illness, WNV2 was first detected in Europe in 2004 in Hungary (10). Following a large outbreak in Greece in 2010, highly pathogenic emergent strains classified within the WNV2a sub-lineage spread rapidly and became dominant in European countries (11). Another sub-lineage, WNV2b, is linked to outbreaks in southern Russia since 2007 and has been sporadically detected in Romania, Italy, and Greece (11). Interestingly, a recent report identified an individual in the United States who was co-infected with both WNV1 and WNV3 (12). Despite WNV3 being primarily isolated from European mosquitoes, this individual had no history of international travel. However, additional surveillance suggests a plausible route for domestic exposure as WNV3 may be circulating at very low levels within United States mosquito populations (13). Overall, this suggests that other divergent, highly pathogenic WNV strains capable of infecting humans can emerge in the United States.

WNV is structured with an asymmetric ribonucleocapsid core (14), consisting of the C protein and RNA, encased in an icosahedral shell of E and prM/M proteins anchored within a lipid bilayer (15). The E protein is critical for infection, facilitating cell attachment and the fusion of viral and cellular membranes. The virus initially buds into the endoplasmic reticulum as immature, non-infectious particles characterized by prM-E trimers (16). As these virions move through the secretory pathway, prM is cleaved into pr and M via acidification, triggering significant surface spike rearrangements (17, 18). Architecturally, the E protein is composed of three domains (EDI, EDII, and EDIII) organized into antiparallel homodimers that form a distinct herringbone pattern. The E protein is the principal antigen that is a target of neutralizing antibodies and the antigen encoded in vaccine candidates (19–21). Over the past two decades, several WNV vaccine candidates have advanced to phase 1 and 2 clinical trials, the majority of which utilize the E protein sequence derived from WNV1 (2). However, WNV1 and WNV2 exhibit more than 30 amino acid differences distributed across the distinct structural domains of the E protein. This degree of structural divergence highlights a significant potential gap in cross-protective immunity between these highly pathogenic WNV strains.

We utilized well-characterized longitudinal and cross-sectional cohorts of WNV RNA-positive blood donors to examine the breadth of the humoral immune response during natural human infection (22). Using a longitudinal cohort of WNV-infected donors from the United States, we found that WNV E protein binding and neutralizing antibodies were rapidly induced and peaked during the acute phase. Interestingly, we observed a poor correlation between binding and neutralization titers during the acute phase, which improved steadily through convalescence. Using a biphasic model to measure decay rates, we estimate the half-life for WNV1 neutralizing antibodies at *t*_1/2_= 37 days for the rapid decay phase and at *t*_1/2_= 472 days for the slow decay phase. The magnitude or persistence of virus, as measured in the plasma or whole blood, largely did not correlate with antibody responses during the acute or convalescent phases of infection. Further, we observed a significant reduction in binding and neutralization titers between WNV1 and WNV2, as measured across the acute or convalescent phases of infection. Lastly, we confirmed the cross-neutralization reduction using an independent cohort of WNV1- or WNV2-infected human donors from Italy and observed similar reductions in heterologous binding and neutralizing antibody responses. These findings demonstrate that WNV1 and WNV2 are divergent lineages that potentially limit cross-lineage immunity.

## RESULTS

### WNV infection generates durable envelope binding and neutralizing antibodies

To characterize the kinetics and durability of the humoral immune response to natural WNV infection, we analyzed an established longitudinal cohort of 55 WNV-infected blood donors identified in the United States between 2009 and 2011 (22). All donors were screened and enrolled following two independent WNV-confirmed tests on plasma samples using an RNA transcription-mediated amplification assay (index). In total, 360 plasma samples were evaluated, with the follow-up extending up to 391 days from the index donation.

**Fig 1:**
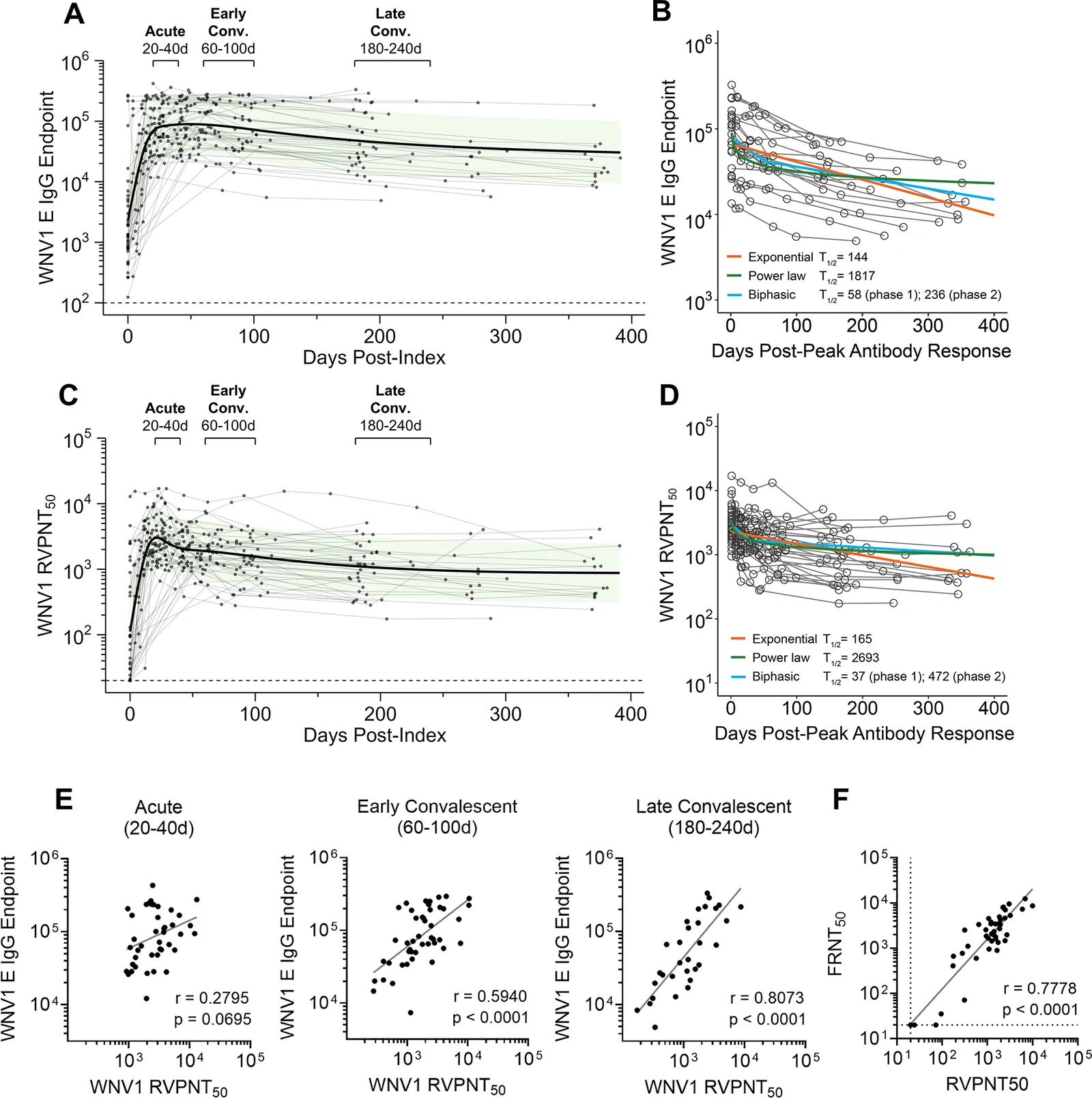
Time course and correlation of the neutralizing and binding antibody response against WNV1. (**A**) Total IgG-binding antibody titers against the WNV1-NY99 strain of West Nile virus between index donation and up to 391 days after (*n* = 55 donors). The solid line shows the population mean trajectory from a spline-based mixed-effect model (5df; random intercept per donor), the shaded band represents ± 1 total marginal SD, the thin gray lines connect repeated measurements within donors, and the dashed line represents the limit of detection. (**B**) Binding antibody decay curves fitted with exponential, power-law, and biphasic (piecewise exponential) models. (**C**) Neutralizing antibody titers, shown as RVPNT_50_ (the reciprocal dilution of plasma that neutralizes 50% of input RVP), against the WNV1-NY99 strain of West Nile virus between index donation and up to 391 days after (*n* = 55 donors). The solid line shows the population mean trajectory from a spline-based mixed-effect model (5df; random intercept per donor), the shaded band represents ±1 total marginal SD, the thin gray lines connect repeated measurements within donors, and the dashed line represents the limit of detection. (**D**) Neutralizing antibody decay curves fitted with exponential, power-law, and biphasic (piecewise exponential) models. (**E**) Spearman correlation plots between WNV1 total IgG binding and neutralization titers at acute (20–40 days, *n* = 43 donors), early convalescent (60–100 days, *n* = 47 donors), and late convalescent (180–240 days, *n* = 31 donors) phases. (**F**) Spearman correlation plot between WNV1 RVPNT_50_ and WNV1 live virus FRNT_50_. The dashed line represents the limit of detection.

We measured envelope-specific IgG-binding titers using an indirect enzyme-linked immunosorbent assay (ELISA) with WNV envelope monomers as the antigen bait. We found that nearly every donor showed a rapid rise in envelope-binding IgG antibodies between the initial index time point and 40 days post-index. While the estimated population mean trajectory showed a consistent rise and decay, we observed considerable donor-to-donor variance around this mean, with a geometric standard deviation of approximately 3.3-fold (Fig. 1A). Decay rates were calculated to determine the durability of WNV E IgG-binding antibodies (Fig. 1B). We identified individual peak antibody levels within 60 days of the initial visit and aligned trajectories to the peak to characterize post-peak decay. Individuals lacking measurements within this window or with less than 6 months of follow-up were excluded. We fit exponential, power-law, and biphasic (piecewise exponential) mixed-effect models to the aligned data. The half-life for WNV1 IgG-binding antibodies was *t*_1/2_= 1,817 days (95% CI: [1,190, 3,182]) under the power law decay model (estimated at day 90 after the peak) and 144 days (95% CI: [129, 164]) under the exponential decay model. The biphasic decay model partitions the post-vaccination period into two phases, representing antibody decline driven by short-lived plasma cells (phase 1, rapid decay) and long-lived plasma cells (phase 2, slow decay). The biphasic decay half-life for WNV E IgG-binding antibodies was *t*_1/2_= 58 days (95% CI: [40, 106]) for the rapid decay phase and was *t*_1/2_= 236 days (95% CI: [187, 320]) for the slow decay phase.

We next performed neutralization assays using a reporter virus particle (RVP)-based neutralization test (Fig. 1C). This tractable system allows for rapid manipulation of structural genes that encode variants, production of high-titer RVPs, and rapid evaluation of antibody neutralization at BSL2 (23). The RVP system has been validated in the literature and shown to perform similarly to the live virus plaque reduction neutralization test (24). Additionally, we found a similar performance between the RVP system and a live virus focus reduction neutralization test (Fig. 1F). Like our observations with envelope protein binding assays, we found that WNV-neutralizing antibodies showed a rapid rise in titers from the initial index visit through 40 days post-index in nearly every donor. Individual neutralizing trajectories similarly displayed substantial variance around the population mean, with an overall geometric standard deviation of roughly 2.8-fold (Fig. 1C). In a similar manner to the binding assays, we identified individual peak antibody levels within 60 days of the initial visit, aligned trajectories to the peak to characterize post-peak decay, and fitted three mixed-effect models to the aligned data (Fig. 1D). The half-life for WNV1-neutralizing antibodies was *t*_1/2_= 2,693 days (95% CI: [1,456, 6,713]) under the power law decay model (estimated at day 90 after the peak) and 165 days (95% CI: [133, 219]) under the exponential decay model. The biphasic decay half-life for WNV-neutralizing antibodies was *t*_1/2_= 37 days (95% CI: [26, 62]) for the rapid decay phase and 472 days (95% CI: [256, 3,049]) for the slow decay phase.

To account for variable sample collection intervals, we stratified the longitudinal data into three immunological phases: acute (20–40 days post-index), early convalescent (60–100 days), and late convalescent (180–240 days). Spearman’s correlation analysis (Fig. 1E) revealed that the relationship between binding and neutralizing antibody titers was initially weak during the acute phase (*r* = 0.2795) but strengthened substantially during the early (*r* = 0.5940) and late (*r* = 0.8073) convalescent phases. Together, these data indicate that human WNV infection elicits a rapid and durable humoral response, with the functional quality of IgG-binding antibodies continuing to mature over 6–12 months to increasingly correlate with neutralization capacity.

### Persistence of WNV RNA in blood or plasma does not influence the magnitude of the antibody response

**Fig 2:**
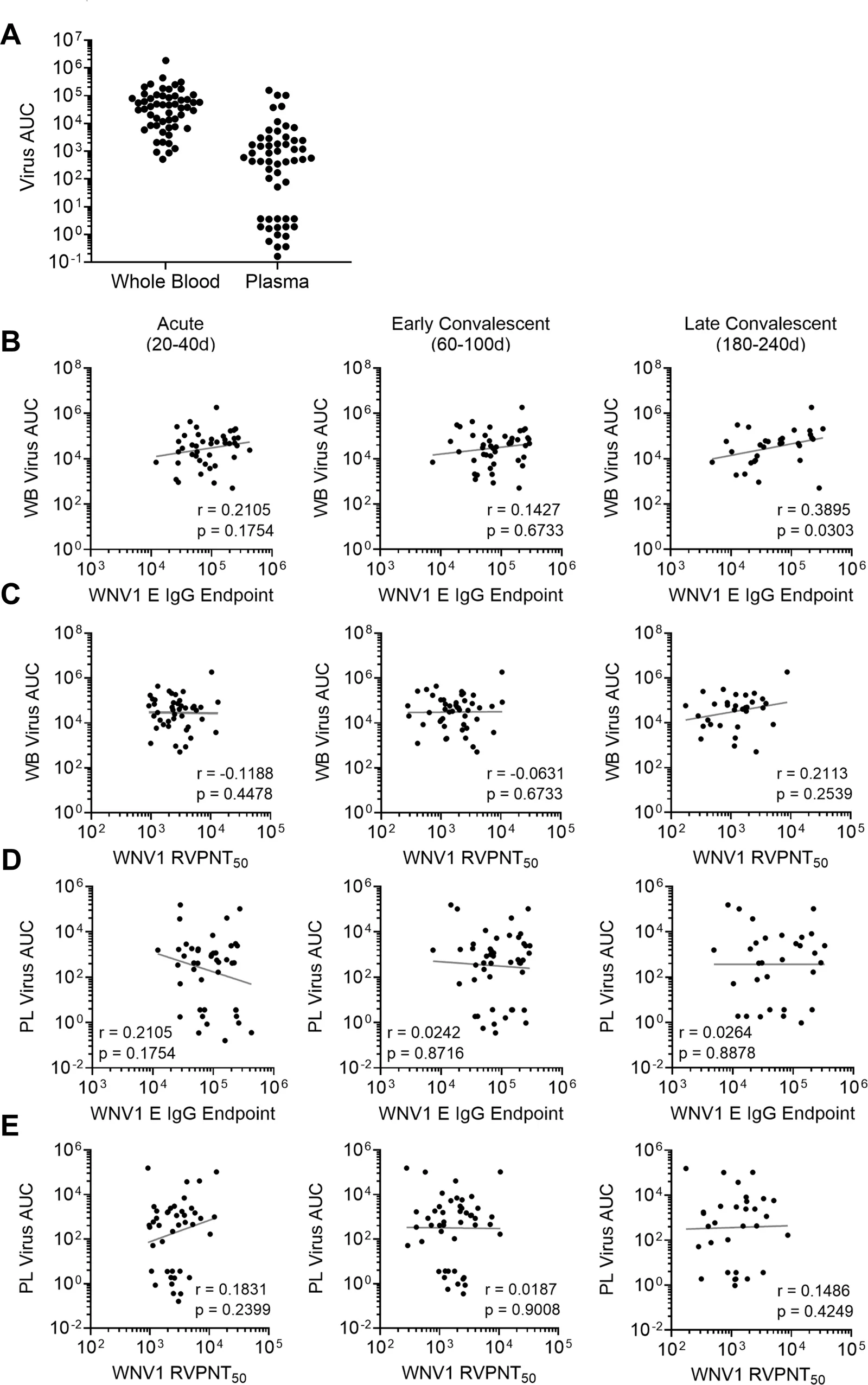
Correlation between WNV viral load and both neutralizing and binding antibody responses against WNV1. (**A**) Plots of the area under the curve (AUC) of WNV viral RNA quantity over 391 days post-index in whole blood (left) and plasma (right) from all donors (*n* = 55). Spearman correlation plots between (**B**) whole blood (WB) virus AUC and WNV1-binding titers, (**C**) WB virus AUC and WNV1-neutralizing titers, (**D**) plasma (PL) virus AUC and WNV1-binding titers, and (**E**) PL virus AUC and WNV1-neutralizing titers. These values are shown at acute (20–40 days post-index, *n* = 43 donors), early convalescent (60–100 days post-index, *n* = 47 donors), and late convalescent (180–240 days post-index, *n* = 31 donors).

Previous studies have shown that WNV RNA in plasma samples becomes undetectable by nearly 2 weeks post-index (25). However, using whole blood samples collected from this 2009–2011 cohort, Lanteri et al. found that, in over 40% of donors, WNV RNA persisted in the red blood cell faction in whole blood for up to 3 months post-index (22). Furthermore, the magnitude of WNV RNA persistence in whole blood directly correlated with the initial viral load present in the plasma (22). We next evaluated whether persistence of viral RNA influenced antibody magnitude and duration. Using area under the curve (AUC), we measured the cumulative persistence of WNV RNA in either plasma or whole blood compartments based on previously published PCR quantification (23). In Fig. 2A, we show the distribution of virus AUC in whole blood and plasma, which agrees with previously published observations about this cohort that detectable WNV RNA varies in magnitude (22). There are two distinct groups in the plasma virus AUC data, with one group appearing separate due to lack of plasma viral load detection. In the plasma and whole blood compartments, we generally observed no significant correlation between WNV binding (Fig. 2B and D) or neutralizing titers (Fig. 2C and E) and the duration of viral RNA persistence across the acute and early convalescent phases. We did note a modest, yet statistically significant, correlation between whole blood viral AUC and WNV1 E IgG titers during the late convalescent phase (180–240 days post-index). Nevertheless, these data collectively indicate that the persistence of viral RNA within the blood does not appear to significantly influence the magnitude or durability of the antibody response to WNV.

### WNV antibodies show reduced binding and neutralizing activity against heterologous WNV strains

**Fig 3:**
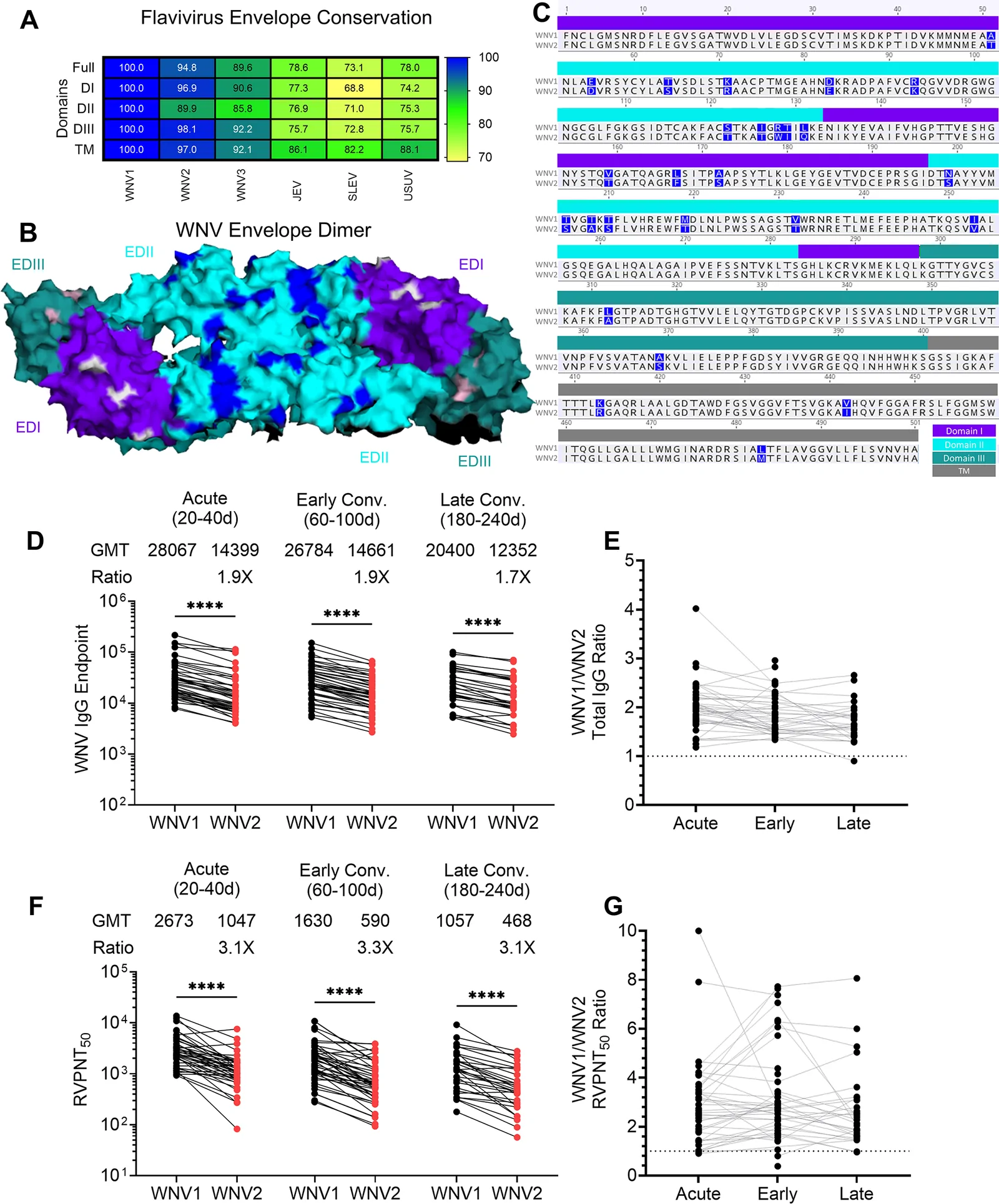
Neutralizing antibody activity from WNV1-infected donors in the United States against WNV1 compared to WNV2. (**A**) A table representing the percent homology within the E protein amino acid sequence of WNV1, WNV2, WNV3, JEV, SLEV, and USUV compared to WNV1. (**B**) The WNV E protein dimer highlighting ectodomain I (purple) and its mutations (gray), ectodomain II (light blue) and its mutations (blue), and ectodomain III (teal) and its mutations (pink). (**C**) Amino acid sequence alignment between WNV strains WNV1-NY99 (GenBank: NC_009942.1) and WNV2-21RS2511 (GenBank: OP009517.1) used for WNV E protein and RVP generation. (**D**) The binding activity against WNV1 and WNV2 E protein shown as total IgG endpoint titer along with geometric mean titer (GMT) and the mean fold change between matched samples against WNV1 and WNV2. (**E**) Matched-pair plot of the fold change between WNV1- and WNV2-binding titers. (**F**) The neutralization activity against WNV1 and WNV2 shown as RVPNT_50_ (the reciprocal dilution of plasma that neutralizes 50% of input RVP) along with GMT and the mean fold change between matched samples against WNV1 and WNV2. (**G**) Matched-pair plot of the fold change between WNV1- and WNV2-neutralizing titers. These values are shown at acute (20–40 days post-index, *n* = 43 donors), early convalescent (60–100 days post-index, *n* = 47 donors), and late convalescent (180–240 days post-index, *n* = 31 donors). Lines connecting between dots represent matched plasma samples. Dashed lines in panels E and G represent a fold change of 1. Statistical significance between matched samples was determined using a Wilcoxon matched-pair signed rank test (*P < 0.05, **P < 0.01, ***P < 0.001, and ****P < 0.0001).

We next compared the E protein sequence of two representative lineage 1 and lineage 2 WNV strains. We compared the prototypic lineage 1 WNV NY99 (WNV1-NY99) with Italy/2021/Padova/21RS2511 (lineage 2; WNV2-21RS2511), a lineage 2 strain isolated in Italy in 2021 (26). We identified 26 amino acid changes, corresponding to a difference of 5.1%, within the envelope protein distributed across ectodomains I, II, and III (Fig. 3A and C). Most of these changes were located within domain II, which is a key target site for antibodies in humans (27). We mapped these residues onto the structure of the WNV E protein dimer and found that several mutations were located at the dimer interface (Fig. 3B).

We next evaluated the effects on binding and neutralization activity of plasma samples from the U.S. 2009–2011 cohort using WNV1 and WNV2 envelope proteins and RVPs. Across the acute and convalescent phases, we observed significant reductions in binding of WNV1 sera to WNV2 envelope protein (Fig. 3D and E). We observed donor-to-donor variability in reductions in binding during the acute (1.9-fold [1.2- to 4.0-fold]), early convalescent (1.9-fold; [1.3- to 3.0-fold]), and late convalescent phases (1.7-fold [0.9- to 2.7-fold]). In terms of neutralizing activity, we observed an increased donor-to-donor variability and greater reduction in neutralization titers between WNV1 and WNV2 (Fig. 3F and G). During the acute phase, we observed a reduction of RVPNT_50_ GMT of 2,673 for WNV1 to 1,047 for WNV2 (3.1-fold [0.9- to 10-fold]). Similarly, during early convalescence, we observed a reduction of RVPNT_50_ GMT of 1,630 for WNV1 to 590 for WNV2 (3.3-fold; [0.8- to 7.7-fold]), and during late convalescent, we observed a reduction of RVPNT_50_ GMT of 1,057 for WNV1 to 468 for WNV2 (3.1-fold; [1.0- to 8.1-fold]). These findings demonstrate that antibodies elicited by WNV1 infection have reduced neutralizing potency against WNV2 compared to WNV1 and that these reductions are highly variable between donors.

**Fig 4:**
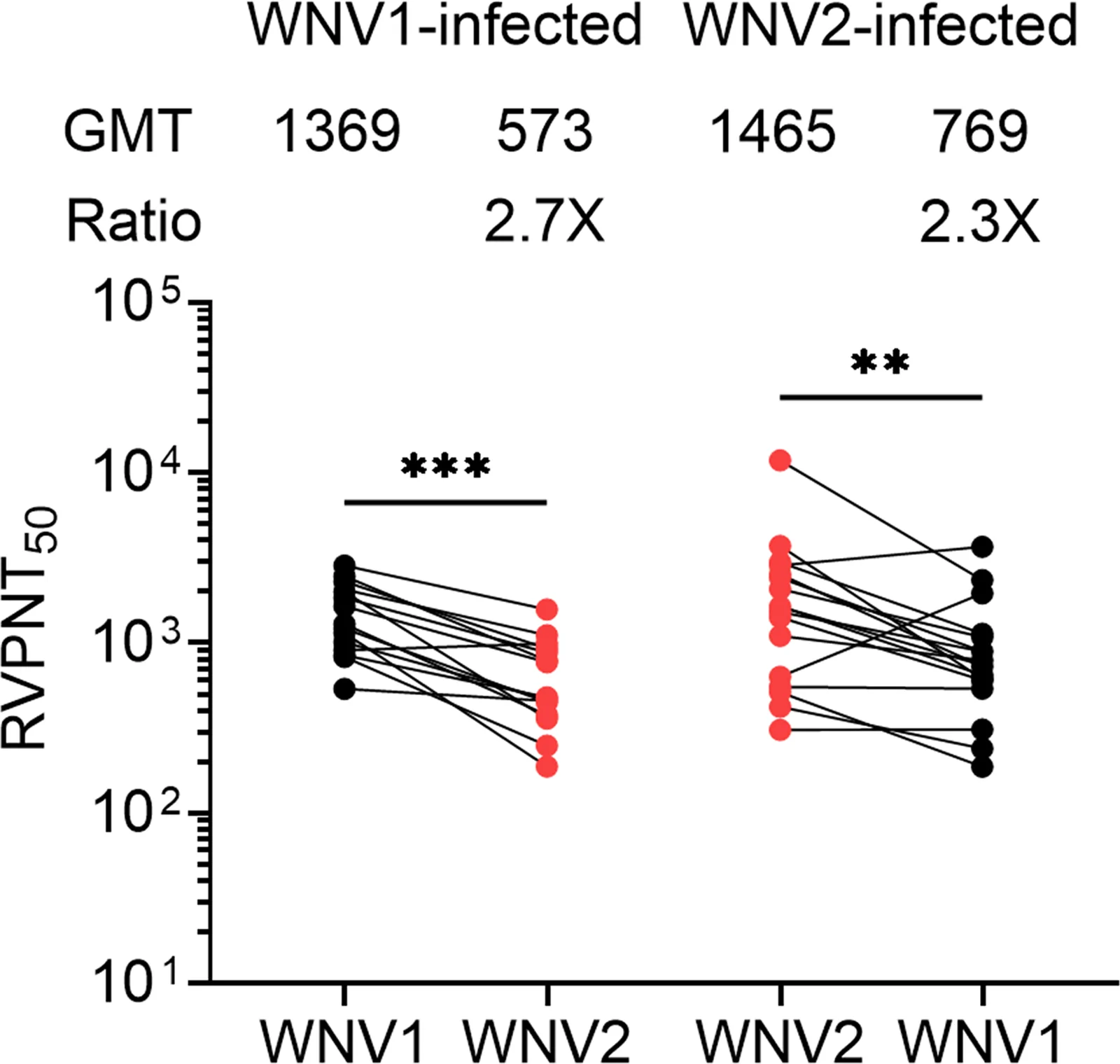
Neutralizing antibody activity from WNV1-infected donors and WNV2-infected donors in Italy against WNV1 and WNV2. (Left) Neutralization activity of WNV1-infected donor plasma (*n* = 15) against WNV1 compared to WNV2. (Right) Neutralization activity of WNV2-infected donor plasma (*n* = 17) against WNV2 compared to WNV1. Neutralization activity is shown as RVPNT_50_ (the reciprocal dilution of plasma that neutralizes 50% of input RVP) along with geometric mean titer (GMT) and fold change. Lines connecting between dots represent matched plasma samples. Statistical significance between matched samples was determined using a Wilcoxon matched-pair signed rank test (*P < 0.05, **P < 0.01, ***P < 0.001, and ****P < 0.0001).

To confirm these findings, we evaluated heterologous neutralizing activity using an independent cohort of WNV-infected individuals from Italy. This cohort included 33 donors, comprising 16 WNV1-infected and 17 WNV2-infected individuals, with clinical infections confirmed and distinguished by real-time RT-PCR. Similar to our US cohort, we found that antibody titers to the infecting lineage were substantially higher than those against the other lineage. Thus, among WNV1-infected donors (Fig. 4), we observed a reduction of RVPNT_50_ GMT of 1,369 for WNV1 to 573 for WNV2 (2.7-fold; [0.9- to 6-fold]). Similarly, among WNV2-infected donors, we observed a reduction of RVPNT_50_ GMT of 1,465 for WNV2 to 769 for WNV1 (2.3-fold; [0.3- to 6-fold]). By demonstrating reciprocal deficits in cross-neutralization between WNV1 and WNV2, these findings validate our observations in the United States cohort and highlight cross-lineage vulnerabilities.

## DISCUSSION

In this study, we demonstrate that natural infection with WNV1 elicits a robust and highly durable humoral immune response, though it exhibits significant vulnerabilities to heterologous viral strains. By longitudinally tracking a cohort of 55 infected blood donors up to 1 year, we found that WNV-specific neutralizing antibodies exhibit decay kinetics consistent with a biphasic decay model, with an initial rapid phase (half-life, 37 days) followed by a slower phase (half-life, 472 days). Notably, the magnitude and persistence of these responses were independent of viral RNA persistence in the whole blood compartment. Despite this durable homologous immunity, we observed a marked reduction in cross-neutralizing activity against the divergent WNV lineage 2 during both the acute and convalescent phases. This deficit was reciprocally confirmed in an independent cohort from Italy, underscoring that genetic divergence between WNV lineages presents a risk of evading pre-existing neutralizing antibodies.

Natural WNV infection triggers a rapid humoral response that is essential for controlling acute viremia and establishing durable protective immunity. In humans, the appearance of WNV-specific IgM closely follows initial viral RNA detection, with a median of 3.9 days. IgG seroconversion begins shortly thereafter, with a median of 7.7 days following initial RNA detection (25). The clearance of circulating virus from the plasma corresponds directly with the surge of these early antibodies as WNV RNA levels typically fall below detection limits at a median of 13.2 days (25). These data suggest that while humoral immunity is a major contributor to controlling viremia, murine models have consistently demonstrated that innate and T cell-mediated immunity are also vital for overall protection (28). Our findings align with these observations as we detect a rapid surge in neutralizing IgG during the acute phase of infection. Murine models confirm that these rapid kinetics are essential for survival. Mice deficient in B cells result in more than a 500-fold increase in viral load in the central nervous system and uniformly fatal infections even at low viral doses (29). Specifically, the early secretion of neutralizing IgM is required to sufficiently control early viral replication, and the magnitude of IgM titers by day 4 post-infection is highly predictive of survival outcomes (30). Previous studies have shown that WNV-specific IgM antibodies are detectable for up to 1 year post-infection (25, 31, 32). These observations demonstrate that the rapid release of virus-specific IgM and IgG antibodies is a critical barrier to protecting against lethal infection by preventing dissemination into the central nervous system. Future studies should focus on evaluating the durability of WNV-specific neutralizing IgM antibodies and characterizing the magnitude and durability of IgG subclasses over time.

In many cases of natural WNV infection, detectable viral RNA persists long after acute viremia resolves (22). Consistent with prior observations of blood compartment dynamics, WNV RNA is rapidly cleared from plasma as the early antibody response peaks (22). It remains detectable within the red blood cell (RBC) fraction of whole blood for up to 3 months post-infection, with the highest sustained viral loads in whole blood observed in donors who experienced the highest initial viral loads in plasma (22). This phenomenon is not unique to WNV as a similar dynamic has been extensively documented following Zika virus (ZIKV) infection, where viral RNA remains detectable in the RBC fraction for months after clearance from the plasma (33). The precise nature of this persistent signal remains incompletely defined, and it is not clear whether the detected viral RNA represents an intact antigen. WNV particles have been demonstrated to adhere to the surface of RBCs, where they may remain intact (34). Other flaviviruses, like ZIKV and dengue virus (DENV), have been shown to infect erythroid progenitor cells, which differentiate into mature, non-permissive RBCs and trap virus particles inside (35, 36). It remains unclear whether these particles are infectious as none have been recovered from donors with persistent RNA detection. This sustained presence led us to hypothesize that prolonged antigen exposure could continuously stimulate the immune system, thereby driving ongoing affinity maturation and durable antibody maintenance. However, our current data do not appear to support a relationship between persistent viral burden and enhanced humoral immunity. We largely observed no obvious correlation between the cumulative WNV viral load in either the whole blood or plasma compartments and the magnitude or neutralizing potency of the ensuing antibody response. These findings suggest that the persistent WNV RNA is effectively compartmentalized within the RBC fraction. If this viral RNA is contained within virus particles, these are sequestered from the immune system, restricting further immunogenicity.

Our evaluation of the cross-reactive potency of WNV1-elicited antibodies against WNV2 highlights the nuances of cross-protective immunity posed by antigenic diversity between emerging viral lineages. The E protein of WNV2-21RS2511 differs from that of WNV1-NY99 by 26 amino acids, representing an overall homology of 94.8%. Our findings show that this modest degree of sequence variation is sufficient to reduce cross-lineage neutralization, with a mean decrease in neutralizing titers between 3.1- and 3.3-fold across the acute and convalescent phases. We also observed high donor-to-donor variability, with reductions spanning from 1 to 10-fold against WNV2 among specific individuals. Because there are currently no known correlates of protection for WNV in humans, it remains difficult to state how this *in vitro* reduction impacts *in vivo* protection from infection, disease, or death (37).

Virus neutralization occurs when a sufficient threshold of neutralizing antibodies are bound to the virion envelope protein, which does not always correlate with *in vitro* antibody titers (38). For instance, phase 3 trials of a dengue virus (DENV) vaccine demonstrated discordance between robust vaccine-elicited neutralizing antibodies and vaccine efficacy (39). Further, two amino acid substitutions in the DENV1 envelope protein are sufficient to disrupt neutralization by DENV1 vaccine-elicited immune sera (40). In other flaviviruses, *in vitro* neutralization reductions are linked with *in vivo* outcomes after vaccination. Antibodies elicited by the 17D yellow fever virus (YFV) vaccine in humans exhibit a 10-fold reduction in neutralization against the emerging ES-504 strain, which has been sufficient to cause breakthrough infections (41). Similarly, mice vaccinated against genotype 3 Japanese encephalitis virus (JEV) exhibit only partial protection against an emerging genotype 1 strain despite only a 2-fold reduction in neutralizing antibody titers (42). While our results show that WNV cross-lineage neutralization can differ by up to 10-fold in some individuals after natural infection, it is unclear to what extent this poses a threat to protective immunity as reinfection with WNV has never been documented and there are no human vaccines. These findings throughout other flaviviruses suggest that these individuals may face a risk of reduced cross-protection. However, the dynamics between cross-neutralization and cross-protection are complex. For example, while prior DENV infection can elicit limited *in vitro* cross-neutralization against ZIKV, it has also been demonstrated to confer cross-protection *in vivo* (43, 44). While many flavivirus cross-reactive antibodies are poorly neutralizing, there are potently neutralizing antibodies that target quaternary epitopes between flavivirus envelope proteins (45). Future studies of epitope footprinting within the WNV envelope protein will provide a better understanding of lineage-specific and cross-reactive antibody responses in humans to contextualize these neutralization differences (46).

Overall, our findings demonstrate that WNV1 infection generates a rapid and robust antibody response. This response is highly durable, with low decay rates, and provides potent neutralization throughout convalescence. We further demonstrate that the persistence of viral RNA in whole blood largely does not impact the magnitude or durability of the sustained antibody response. Crucially, the potent WNV1-elicited antibody responses showed significantly reduced cross-neutralization of WNV2. This finding was also validated in a separate longitudinal cohort from Italy, which included both WNV1- and WNV2-infected donors. These findings have a direct impact on vaccine development. Previous WNV vaccine candidates have relied on a prototype vaccine approach, using a single representative isolate such as the WNV1-NY99 strain. The data presented herein suggest that this approach could leave vaccinated populations vulnerable to emerging divergent lineages (2). To overcome these antigenic barriers and create a vaccine that confers broad protection, future WNV vaccine development efforts should prioritize a multivalent antigen design and a vaccine that elicits a robust and durable antibody response.

## MATERIALS AND METHODS

### Study population and plasma samples

United States blood donors confirmed positive for WNV between 2009 and 2011 were enrolled by Blood Systems Research Institute (BSRI), as previously described (22). These donors tested positive for WNV RNA and answered detailed questionnaires at study enrollment as well as 2 weeks later (46). While these donors were not confirmed to be WNV1-infected by type-specific testing, they were confirmed to have not traveled outside of the United States. There is no evidence of strains outside of lineage 1a circulating in North America at the time of the study. Study participants donated additional blood samples up to 1 year after their index donation. Blood was centrifuged at 872 × *g* for 10 min to separate plasma to be aliquoted for long term storage at −80°C (22). For samples from the Italy cohort, blood, urine, and/or cerebrospinal fluid (CSF) samples were collected from subjects with laboratory-confirmed WNV infection, in whom the infecting WNV lineage was determined by specific qRT-PCR assays and/or sequencing. Briefly, total nucleic acids were purified from 200 μL of whole blood, plasma, urine, or CSF by using a MagNA Pure 96 System (Roche Diagnostics, Basel, Switzerland) and were amplified by two in-house real-time RT-PCR methods, which allowed the discrimination between WNV1 and WNV2 (47, 48). Real-time RT-PCR assays were carried out using the one-step real-time RT-PCR kit (Thermo Fisher Scientific, Waltham, MA, USA) and ran on Applied Biosystem QuantStudio 5 thermal cycler (Thermo Fisher Scientific).

### Cells and viruses

VeroE6 cells used for RVPNT assays were cultured in complete Dulbecco’s modified Eagle’s medium (DMEM), supplemented with 10% fetal bovine serum (FBS), L-glutamine, and penicillin-streptomycin and incubated at 37°C with 5% CO_2_. HEK293T cells used for transfection to generate WNV RVPs were in complete DMEM, but with 7% FBS. WNV RVPs were generated using the WNV sub-genomic replicon system engineered to express green fluorescent protein (GFP), as previously described (23). Briefly, HEK293T cells were co-transfected with the WNV replicon plasmid and a complementing structural protein plasmid encoding the capsid, pre-membrane, and envelope genes derived from either the WNV1-NY99 or WNV2-21RS2511 strains. Transfections were performed using Lipofectamine 3000 (Thermo Fisher; L3000008) according to the manufacturer’s instructions. RVP-containing supernatants for the WNV1-NY99 strain were harvested 24 h post-transfection following incubation at 37°C. Supernatants for the WNV2-21RS2511 strain were harvested 96 h post-transfection following incubation at 30°C.

### Generation of WNV E protein

WNV E protein was generated as previously described (49). The expression vectors for the West Nile virus (WNV) New York 1999 (GenBank accession number ABA62343.1, amino acids 1–405) and WNV Lineage 2 (GenBank accession number UVC41610.1, amino acids 1–405) envelope (E) protein were codon-optimized and ordered from Twist Biosciences in the pET21 vector with an N-terminal ribosomal binding site. Both E proteins contain the fusion loop mutants: T76A, M77G, W101R, and L107R (50). Following sequence confirmation by whole-plasmid sequencing (Plasmidsaurus), the construct was transformed into BL21(DE3) cells (New England Biolabs). Briefly, bacteria were grown in Luria-Bertani (LB) broth at 37°C and induced at an optical density at 600 nm (OD600) of ~0.6 with 1.0 mM isopropyl β-D-1-thiogalactopyranoside (IPTG) for 4–6 h prior to harvest by centrifugation. Bacterial pellets were lysed by freeze-thaw followed by sonication in a Tris-EDTA-based buffer, and the E protein was recovered as an insoluble inclusion body fraction by low-speed centrifugation. Inclusion bodies were washed sequentially with detergent-containing and detergent-free buffers to remove membrane and cytosolic contaminants and then resuspended in Tris-EDTA buffer. Inclusion body preparations were solubilized in ~7 M guanidine hydrochloride and ~10 mM β-mercaptoethanol at room temperature, and the insoluble material was removed by centrifugation. The denatured, reduced E protein was refolded by slow pulsed dilution of the solubilized material into a 0.4 M L-arginine refolding buffer containing 100 mM Tris-HCl (pH 8.0), 2 mM EDTA, 5 mM reduced glutathione, 0.5 mM oxidized glutathione, and 0.5 mM phenylmethylsulfonyl fluoride (PMSF), with gentle stirring at 4°C. After overnight refolding, the protein solution was clarified by filtration and concentrated by ultrafiltration. Refolded E protein was purified by size-exclusion chromatography on a Superose 75 (S75) column (Cytiva) equilibrated in 20 mM HEPES pH 7.5, 150 mM NaCl, and 0.01% sodium azide. Fractions corresponding to the expected oligomeric state and apparent molecular weight of the E protein were pooled and stored in 20 mM HEPES (pH 7.5), 150 mM NaCl, and 0.01% sodium azide. The refolding of WNV2-E/FLM was the same as described here, except 0.4 M NDSB-201 was substituted for 0.4 M L-arginine in the refolding buffer.

### WNV E-binding assay

The recombinant WNV E proteins were coated on Nunc Maxisorp ELISA plates (Thermo Scientific; 439454) at a concentration of 1 μg/mL in 100 μL phosphate-buffered saline (PBS) overnight at 4°C. The plates were washed extensively with PBS containing 0.05% Tween-20 (PBS-T) and blocked with PBS-T containing 1% FBS (PBS-T-FBS) for 2 h. Plasma samples were heated to 56°C for 30 min and stored at −20°C before use. Plasma samples with serial 3-fold dilution in PBS-T-FBS were added to the plates and incubated at room temperature for 2 h. The WNV E protein-specific IgG signals were detected by incubating with peroxidase-conjugated anti-human IgG (Jackson ImmunoResearch; 109-036-098) for 1 h. The plates were washed and developed using an o-phenylenediamine substrate (Sigma; P8787) in 0.05 M phosphate-citrate buffer (Sigma Aldrich; P4809) containing 0.012% hydrogen peroxide. The absorbance values were measured at OD490. The endpoint titer for binding was determined as the concentration required to achieve a signal equal to that of the negative-control plasma samples at a 1:100 dilution.

### Reporter virus particle-based neutralization test (RVPNT)

Vero E6 cells were used to seed clear, tissue culture-treated 96-well plates (Greiner Bio-One; 655090) at 3 × 10^4^ cells per well and then incubated at 37°C for 24 h or until the cells reached 100% confluency. The plasma specimens in duplicate were heat-inactivated and diluted serially threefold using serum-free DMEM, from a starting dilution of 1:20 up to a final dilution of 1:43,740. Diluted samples were mixed with an equal volume of WNV RVP (800 infectious units per well) and incubated at 37°C for 1 h. The virus-serum mixtures were added onto the Vero E6 cells and incubated again at 37°C for an additional hour. After incubation, the inoculum was removed and replaced with DMEM. The cells were further incubated for 48 h at 37°C. All media were removed, and the cells were washed with PBS, fixed with 2% paraformaldehyde for 30 min, and washed twice again. Each plate of cells was imaged using the Cytation 7 fluorescent microscope to detect GFP-positive cells, indicating an RVP infectious unit.

### Quantification and statistical analysis

Antibody neutralization was quantified by counting the number of GFP-positive cells for each sample at each dilution. RVPNT_50_ titers represent the reciprocal plasma dilution in which the RVP infectious units are reduced by 50% and were interpolated using a 4-parameter nonlinear regression in GraphPad Prism 10.5.0. Samples that do not reach 50% neutralization are plotted at 20, as the limit of detection, for geometric mean calculations. The normality of antibody binding and neutralization titers were determined using the Shapiro-Wilk normality test. Pairwise analysis of antibody binding and neutralization titers was performed by using the Wilcoxon matched-pair signed rank test. The Spearman rank test was used to determine correlation, and these analyses were done by log transforming the binding and neutralization titers. Antibody decay curves were fitted with exponential, power-law, and biphasic (piecewise exponential) using MonolixSuite2024R1.

### Mixed-effect modeling

To summarize the population-level trajectory and spread of each marker over time, we fit linear mixed-effect models with a natural cubic spline of days post-index as a fixed effect (df = 5) and a random intercept for the donor. Spline degrees of freedom were selected by minimizing BIC across a range of complexities (df: 1–8); df = 5 minimized BIC for both IgG endpoint titers and RVPNT50. This approach yields an estimated population-mean trajectory and a total marginal standard deviation, calculated as the square root of the summed between-donor and residual variances, depicting the donor-to-donor spread around the mean at each time point (Fig. 1A and C).

### Sequence homology of flavivirus envelope protein domains

Sequence identities were extracted from the alignments using Geneious 2026.0.2 and accession numbers: NC_009942.1 (WNV1-NY99), OP009517.1 (WNV2-21RS2511-6), PP445212.1 (WNV3-135322a-L3), NC_001437.1 (JEV), NC_007580.2 (SLEV), and NC_006551.1 (USUV). Envelope domain homology was derived from sequence identities, with WNV1-NY-99 used as the reference sequence. Heatmaps were constructed using GraphPad PRISM for Mac OS (version 11.0).

### Structural modeling

WNV envelope dimer structure (PDB: 7KVA) was visualized and manipulated in Pymol (version 3.1.8)

## Data Availability

All data associated with the paper can be made available to academic researchers. Requests should be addressed to corresponding author Mehul S. Suthar.
